# Quantifying Cancer Healthcare Costs for Adolescents and Young Adults in Queensland, Australia

**DOI:** 10.3390/healthcare13243302

**Published:** 2025-12-16

**Authors:** Carla Thamm, Shafkat Jahan, Raymond J. Chan, Gail Garvey

**Affiliations:** 1 Caring Futures Institute, College of Nursing and Health Sciences, Flinders University, Adelaide, SA 5042, Australia; raymond.chan@flinders.edu.au; 2 First Nations Cancer and Wellbeing Research Program, School of Public Health, Faculty of Health, Medicine and Behavioural Sciences, The University of Queensland, Brisbane, QLD 4006, Australia; shafkat.jahan@uq.edu.au (S.J.); g.garvey@uq.edu.au (G.G.)

**Keywords:** health service costs, health service use, adolescent and young adult, cancer, treatment, out-of-pocket costs

## Abstract

**Highlights:**

**What are the main findings?**

- A diagnosis of cancer between the age of 15–24 imposes significant economic and human costs for the healthcare system, the cancer survivor and their family.
- Costs were higher for public hospital vs. private, with service use peaking in the first six months post-diagnosis and varied by cancer type and sociodemographic factors.

**What are the implications of the main findings?**

- There is a need for targeted, cost-effective models of care to support long-term survivorship in this population.
- Sociodemographic characteristics should be considered when targeting models of care.

**Abstract:**

Background: Adolescents and young adults (AYAs) with cancer have unique needs as they transition from childhood to adulthood. This study explored the patterns of health service use and the related costs incurred by the health care system and out-of-pocket (OOP) costs for AYAs diagnosed with cancer in Queensland, Australia. Methods: A linked administrative dataset (CancerCostMod) containing all AYA cancer survivors (*n* = 871; aged 15–24) diagnosed between July 2011 and June 2015 from the Queensland Cancer Registry (QCR) linked these records to Queensland Health Admitted Patient Data Collection (QHAPDC), Emergency Department Information System (EDIS), Medicare Benefits Schedule (MBS), and Pharmaceutical Benefits Scheme (PBS) records from July 2011 to June 2018. We quantified total and average health service use, associated costs, OOP costs, and costs variations across sociodemographic characteristics. Results: The public hospital costs incurred for AYAs diagnosed with cancer were higher (AUD 33.7 M) compared to private hospitals (AUD 12.6 M). The median cost per person for public admissions (AUD 9759, IQR = AUD 0–37,245). AYAs claimed 215,900 MBS items and 58,321 PBS items over a five-year period. First Nations Australians and individuals living in regional and mostly disadvantaged areas had higher public hospital admissions, associated costs, and ED admissions compared to their counterparts. Conclusions: This study revealed significant variations in healthcare costs for AYAs diagnosed with cancer. Public hospital costs were higher, with notable differences observed across sociodemographic characteristics.

## 1. Introduction

Adolescents and young adults (AYAs) with cancer are a heterogenous population with unique needs as they transition from childhood to adulthood [1,2]. In 2022, approximately 1.3 million new cancer cases and 377,621 cancer-related deaths occurred among AYAs worldwide, with an age-standardised incidence rate (ASIR) of 40.3 per 100,000 and an age-standardised mortality rate (ASMR) of 11.8 per 100,000 [3]. In Australia, AYAs are defined as 15–24 years at diagnosis [2]. Approximately 5300 new cancers were diagnosed in the AYA age group in Australia between 2014 and 2018, with this population having a five-year relative survival rate of 90% from all cancers combined [2].

The economic and human costs of receiving a cancer diagnosis at this stage in life are substantial for the healthcare system, the AYA cancer survivor, and their family [4,5]. The costs associated with cancer can vary substantially from diagnosis through to treatment and beyond [6]. Despite the high survival rates, these costs pose significant challenges to both patients and the broader healthcare system, particularly given that AYAs often face the added burden of long-term health issues resulting from cancer treatment. In 2016, it was estimated that the total lifetime costs for an individual diagnosed with cancer between the ages of 15 and 25 in Australia were AUD 1.4 billion (including burden of disease, health and informal care costs, and productivity losses) [5]. Hospital admissions was the largest component of costs, followed by out of hospital services expenses and pharmaceuticals costs [5]. Remoteness, socioeconomic status, Indigenous status, and cancer type have also been shown to influence the cost of treatment [2,5].

Care for AYA cancer survivors is complex and can be challenging because they experience cancers that are common in paediatric patients (e.g., central nervous system [CNS] cancers), as well as those common in older adults (e.g., breast cancer), and are often in a transition phase between paediatric and adult hospital settings [1]. Furthermore, some cancers are more prevalent in this age group than in others, such as Hodgkin lymphoma and gonadal germ cell tumours [1]. The incidence of melanoma is also high in this age group in Australia compared to global data [2].

A Canadian study identified that AYA cancer patients treated in paediatric centres used more healthcare resources and incurred higher costs than those treated in adult settings [7]. Decisions about where these patients receive care is often driven by funders and can impact the development of models of care. Australia’s healthcare system is a multifaceted mix of public and private providers funded by various federal, state, and territory governments, as well as private health insurers and individuals [8]. The Australian, state, and territory governments, as well as the non-government sector, also share responsibility for the management and regulation of this complex health system [8].

Currently there is limited understanding of the service usage of AYAs with cancer and the costs to funders and individuals, or how these differ for those from diverse sociodemographic backgrounds. Therefore, the aim of this study was to utilise linked administrative data to explore the patterns of health service use and associated costs to the healthcare systems, as well out-of-pocket (OOP) expenses, over five years following a cancer diagnosis for AYAs in Queensland, Australia. By incorporating OOP costs, this study offers a comprehensive understanding of the financial burden faced by both AYA patients and the healthcare system. We also conducted a subgroup analysis to investigate the health service use and cost variations across different demographic characteristics and cancer types. This information can be used by healthcare funders and leaders to guide personalised and cost-effective models of care.

## 2. Materials and Methods

### 2.1. Study Design and Population

This study investigated the costs of cancer in AYAs from the perspective of the healthcare service using CancerCostMod, a linked administrative dataset [9,10]. This Australian population-based dataset includes all patients diagnosed with cancer in Queensland (except non-melanoma skin cancer) reported by the Queensland Cancer Registry (QCR) from 1 July 2011 to 30 June 2015 (*n* = 106,571). Individual records from the QCR were linked to Queensland Health Admitted Patient Data Collection (QHAPDC), Queensland Health Emergency Department Information System (EDIS), Medicare Benefits Schedule (MBS), and Pharmaceutical Benefits Scheme (PBS) records from 1 July 2011 to 30 June 2018 that provided a minimum of three years of follow-up after diagnosis. This study focused on a specific subgroup within the CancerCostMod population, namely AYAs aged 15–24 who had been diagnosed with cancer. This resulted in a final study sample size of 871 individuals.

The QCR database contains information on cancer type (ICD-O codes) and sociodemographic characteristics at the time of cancer diagnosis, such as age, sex, Indigenous status, and residential postcode. Indigenous status was available for 88% of the original QCR dataset. Multiple imputation techniques were employed to impute the missing data points for Indigenous status. Postcodes were used to determine the Index of Relative Socioeconomic Disadvantage (IRSD), which summarises the economic and social conditions of an area. This index was categorised into quintiles, Q1 representing the most disadvantaged and Q5 representing the least disadvantaged regions. Additionally, the Australian Statistical Geography Standard (ASGS) was used to assess remoteness, classifying areas into metropolitan, regional (inner and outer), and remote (remote and very remote) categories [11].

ICD_10 codes reported in the QHAPDC dataset were mapped to the Charlson Comorbidity Index (CCI) to create a weighted score for patient comorbidities [12].

The MBS and PBS datasets include information such as date of service/prescription, item code, and charge and government rebate for all MBS services and PBS prescriptions accessed within the targeted timeframe. OOP costs for MBS services and prescription claims were calculated as the difference between the charge for the service or prescription and the government rebate received.

### 2.2. Ethics and Consent

Human Research Ethics approval was obtained from the Townsville Hospital and Health Service Human Research Ethics Committee (HREC) (HREC/16/QTHS/11), the Australian Institute of Health and Welfare (EO2017/1/343), James Cook University HREC (H6678), and The University of Queensland HREC (2022/HE002538). Permission to waive individual consent was granted by Queensland Health under the Public Health Act 2005.

### 2.3. Assigning Costs

Costs are presented from the perspective of the healthcare system, including public and private hospital care and emergency department visits. OOP costs for patients, comprising MBS and PBS co-payments, are also reported separately to capture the financial expenses incurred by patients. The expenses associated with each episode of care in public hospitals were assigned to the corresponding Australian Refined Diagnostic-Related Group (AR-DRG) based on the costs reported by the National Hospital Cost Data Collection (NHCDC) for the relevant year. Adjustments were made to account for potential variations in healthcare costs related to patient demographics. For private hospital separations, we determined the cost for each AR-DRG using the average charge per separation specified in the Private Hospital Data Bureau (PHDB) annual reports. Emergency Department (ED) visits were classified using the Urgency Related Group (URG) system, considering triage category, discharge destination, and the primary reason for the visit (ICD-10-AM). The cost assigned to each URG for each ED presentation was based on the average cost per visit reported in the NHCDC report for the relevant year. To focus on episodes potentially related to cancer treatment, we only considered hospital and ED presentations occurring after a cancer diagnosis. Because it was not possible to differentiate whether the admission was specifically related to the cancer diagnosis, all hospital episodes and ED presentations post-diagnosis were included in the analysis. We presented the results of hospital episodes and ED presentations separately due to the relatively recent introduction of the ED classification system. The cost of hospital episodes and ED presentations was calculated monthly for each individual from the date of diagnosis (t = 0) up to 84 months post-diagnosis. If an individual did not utilise any healthcare services during a specific month, a cost of AUD 0 was recorded. All costs were adjusted to 2020 Australian dollars using the Australian Institute of Health and Welfare (AIHW) Health Price Index, which applies annual inflation rates ranging from approximately 1.66% to 2.01%.

### 2.4. Statistical Analysis

Descriptive analysis was conducted to identify the demographic characteristics of the sample. Health service use (i.e., hospital episodes, ED presentations, Medicare and pharmaceutical claims) and related costs were aggregated per month for each patient. Health service use and associated costs were then aggregated into 6-month time periods from the time of diagnosis to examine the total health service use and total cost within each 6-month period for all participants. The mean cost per patient was calculated by dividing the total costs for each period by the number of patients within the relevant patient cohort. Given the skewed distribution of health service use and cost data, between-group comparisons (melanoma vs. other cancers and across sociodemographic and clinical characteristics) were conducted using non-parametric tests. Wilcoxon rank-sum tests and Kruskal–Wallis tests were used to assess differences in median per-person health service use and costs.

A sensitivity analysis was performed by restricting the sample to patients with non-zero health service use over the study period. This analysis assessed whether observed group differences were consistent when participants with zero utilisation were excluded. All analyses were performed using SAS V9.4 [13].

## 3. Results

Between 1 July 2011 and 30 June 2013, 851 AYAs (15- to 24-year-olds) were diagnosed with cancer in Queensland, with a mean age of 20.3 years. The majority (90%) of these patients lived five or more years post-diagnosis. Half (49.5%) lived outside of metropolitan areas. Melanoma was the most common cancer in this population (21.5% of all diagnoses), followed by colon cancer (11.6%), Hodgkin lymphoma (10.5%), and testicular (9.5%) and thyroid (8.5%) cancers. Table 1 presents the demographic characteristics of the AYA cancer survivors at diagnosis.

### 3.1. Health Service Use and Associated Costs to Different Funders over Five Years

Over the five-year period post-diagnosis, public hospitals incurred higher costs (AUD 33.7 M) compared to private hospitals (AUS 12.6 M). The median cost per person for public hospital episodes over five years was AUD 9759 (IQR = AUD 0–37,245) compared to AUD 0 (IQR = AUD 0–8889) for private hospital episodes. Despite higher costs in public hospitals, private hospitals recorded more episodes of care (5146 vs. 4731).

There were 3144 emergency department (ED) presentations over five years, with an average of 2 per person (IQR = 0–6). The total cost to healthcare funders from ED presentations was AUD 2.1 M, with a median cost per person of AUD 1146 (IQR = AUD 0–3330). OOP costs for MBS and PBS items totalled AUD 3.2 M and AUD 710,777, respectively. AYAs claimed 215,900 MBS items and 58,321 PBS items over the five-year period.

### 3.2. Patterns and Cost of Care for Melanoma

Melanoma was the most common cancer among AYAs (21.5%). Given its frequent management outside of hospital settings, melanoma-related data were analysed separately to compare service use and costs with other cancers (Table 2). In both melanoma and other cancers, public hospital episodes were the largest contributors to healthcare costs. The average cost per person was consistently higher for public hospital care. MBS claims and associated OOP costs were significantly greater than PBS claims and costs across both groups.

AYAs with melanoma had notably lower episodes and claims, and significantly lower associated costs across all service types—including public hospital episodes, ED presentations, and MBS/PBS co-payments—compared to those with other cancers (Table 3).

### 3.3. Patterns and Cost of Care of All Cancers Except Melanoma

Significant differences in median hospital admissions and costs were observed across several demographic and clinical groups (Table 4). First Nations AYAs had higher median public hospital admissions (5.5 vs. 2; IQR 3–14 vs. 0–5; *p* < 0.05) and associated costs (AUD 38,980 vs. AUD 11,795; IQR AUD 17,882–74,125 vs. AUD 0–38,984; *p* < 0.05) and higher median ED presentations (5 vs. 2; IQR 2–13.5 vs. 0–4; *p* < 0.05) compared with Non-First Nations AYAs. AYAs from metropolitan areas had significantly lower median public hospital admissions and costs than those from remote or regional areas (*p* < 0.05), while remote/very remote patients had higher ED presentations (*p* < 0.05). Patients from the most disadvantaged areas (IRSD Quintiles 1 and 2) had higher median public hospital admissions, ED presentations, and associated costs than those from less disadvantaged areas (Quintiles 4 and 5; *p* < 0.05). Among cancer types, colon cancer patients had the highest ED visits (*p* < 0.05), while Non-Hodgkin lymphoma patients had the highest public hospital and ED costs and median public admissions (*p* < 0.05). No significant differences were observed in private hospital use or associated costs across these groups.

### 3.4. Sensitivity Analysis

Sensitivity analyses restricted the sample to patients with non-zero health service use (Supplementary Tables S1 and S2). Median costs and service use were generally higher than in the whole study cohort (Table 3 and Table 4), only reflecting actual users. Despite the higher magnitudes, patterns of differences between melanoma and other cancers and across sociodemographic and clinical characteristics remained consistent, indicating that the observed group differences are robust and not driven solely by patients with zero utilisation.

## 4. Discussion

This study used linked administrative data to examine the healthcare use and associated costs of AYAs diagnosed with cancer in Queensland, Australia. In this population-based study, public hospitals incurred higher costs (AUD 33.7 M) over the five years after diagnosis compared to private hospitals (AUD 12.6 M). We also observed variations in costs across different demographic characteristics (i.e., sex, remoteness, socioeconomic disadvantages, First Nations status).

Internationally, the healthcare costs associated with AYA cancers are reported as substantial [4,14,15]. With the incidence of AYA cancer continuing to rise in Australia [2] and globally [16], costs will continue to grow, increasing the economic burden on healthcare and requiring innovative solutions. In Queensland, AYA cancer care is provided across both paediatric and adult settings, supported by a central team with a limited service capacity [17]. Although our study did not breakdown adult vs. paediatric settings, we did identify that public hospitals bore significantly higher total costs compared to private hospitals, despite private hospitals having more episodes of care. The average costs per patient were notably higher for public hospital episodes, likely a reflection of the more intensive and complex treatments offered in these settings.

We also observed significant average MBS and PBS OOP costs per person, indicating that AYA individuals are frequently accessing services. High cure rates for AYA cancer patients (>80%) in developed countries [18] and long-term survival could impact this, with the long-term consequences of cancer treatments on developing organ systems increasing the risk of chronic disease [19]. Despite Australia having universal coverage OOP costs taking up around 15% of total health expenditure, when combined with private insurance premiums, individuals contribute to 26% of total health expenditure [20]. OOP costs are known to disproportionately impact young people and those with chronic illnesses, along with those at lower incomes [21]. People with lower financial resources have been shown to be at a higher risk of experiencing catastrophic healthcare expenditures (spending 10% or more of household income on healthcare) [21]. These increased expenditures can lead to substantial inequalities, including deceased life expectancy and health outcomes, and barriers in accessing healthcare [22].

Due to their life stage, AYA cancer survivors (particularly older AYAs) are at a higher financial risk of this because they experience financial responsibilities that younger and much older cancer patients do not (e.g., starting careers, mortgages, young family commitments, and repaying student loans) [23]. The financial implications of cancer and the burden of long-term chronic disease throughout survivorship, along with insurance and other health service related barriers, can lead to catastrophic healthcare expenditures and has been shown to impact young cancer survivors’ engagement in surveillance and follow-up and long-term outcomes [24,25]. This underscores the importance of having access to longer-term data to track trends over extended periods, which is crucial for healthcare planning and resource allocation for this group of long-term cancer survivors [15]. Further investigation is needed to understand the implications for patients, specifically lifetime financial burden relating to a cancer diagnosis in this age group [4,5,14,15,26].

Melanoma is the most common cancer diagnosis in this age group in Queensland, which differs from other regions in Australia, where Hodgkin lymphoma is more prevalent [2]. Globally, breast and brain/CNS cancers are most prevalent in this is population [1]. In comparison to other cancers, melanoma is often treated in primary care or private skin facilities. To account for this, we estimated the health service use and costs for melanoma separately from other cancers. Our findings show that melanoma patients incurred notably lower costs and service use compared to other cancers, likely due to its less intensive treatment for early-stage disease. Other AYA cancer patients, particularly in the first 6 months following diagnosis, experience more frequent healthcare episodes and higher costs. International data also report a decline in healthcare costs 6 to 12 months post-diagnosis [1].

Subgroup analyses of different demographic groups and cancer types, excluding melanoma, showed that First Nations Australian AYAs had higher public hospital and ED visit costs, possibly due to geographic, cultural, and systemic barriers that affect timely care [27]. Regional patients had the highest public hospital episodes and costs, whereas metro patients had more private hospital episodes. We also found that individuals residing in remote or very remote areas had a higher mean number of ED presentations and incurred higher costs per person compared to those from metropolitan and regional areas. Similarly, public hospital episodes and ED visits were higher in AYAs from the most disadvantaged areas (Quartiles 1 and 2). This may be related to the limited access to primary care for people living outside metropolitan areas in Australia [28,29], leading to an increased use of emergency services for urgent or chronic care needs, which in turn exacerbates costs. Future service provision models should focus on ED prevention and avoidance strategies, particularly for populations in remote or disadvantaged areas.

Leukaemias have been reported to incur the highest cost both in Australia and internationally for children and adolescents with cancer [15]. However, our study found that colon cancer had the most ED visits in Queensland (*p* < 0.05), while Non-Hodgkin lymphoma patients had the highest public hospital and ED costs and median public admissions (*p* < 0.05). This anomaly may be because Leukaemia did not feature in the top five cancers investigated. Further investigation into the cancer types with the highest associated costs would provide valuable insights for healthcare planning and resource allocation.

### Strengths and Limitations

This study provides a unique five-year analysis of healthcare costs for AYAs with cancer in Queensland, using linked administrative data across hospital, medical, and pharmaceutical services. A key strength is the separation of melanoma from other cancers, recognising its distinct treatment pathways. This approach enhances the relevance of findings for service planning and policy development.

However, several limitations should be acknowledged. The study is descriptive and exploratory in nature, focusing on observed patterns of healthcare use and associated costs. Non-parametric tests were conducted, particularly given the skewed distribution of cost data. Cancer staging could not be assessed due to the absence of routine staging data in the Queensland Cancer Registry, limiting the analysis of cost variations by disease severity. International comparisons are constrained by Australia’s narrower AYA age definition (15–24 years); global studies typically include individuals aged 15–39 years, where cancer incidence peaks in the 35–39 age group. This affects comparability in cancer types, treatment settings, and associated costs. Generalisability is further limited by differences in healthcare systems, service volumes, pricing, and policy contexts across jurisdictions. Finally, while direct OOP costs for medical services and pharmaceuticals were captured, indirect costs, such as travel, accommodation, and lost productivity were not, potentially underestimating the total financial burden.

## 5. Conclusions

Healthcare quality has important implications for AYA cancer patients who have a high long-term survival. It is important to consider healthcare utilisation and the costs of cancer for various groups to create sustainable models of care and target appropriate time points so that mechanisms can be put in place to maximise the value for money and reduce wastage in an already strained healthcare system.

## Figures and Tables

**Table 1:** Demographic characteristics of adolescents and young adults with cancer diagnosed in Queensland between 1 July 2011 and 30 June 2015 (*n* = 851).

Variables	*n* (%)
Mean age	20.27
**Mortality**	
Died	79 (9.28)
Alive	772 (90.72)
**Sex**	
Male	409 (48.06)
Female	442 (51.94)
**First Nations Australian Status**	
Non-First Nations Australian	809 (95.06)
First Nations Australian	35 (4.11)
**Remoteness**	
Metropolitan	423 (49.71)
Regional (inner and outer regional)	361 (42.42)
Remote (remote and very remote)	60 (7.05)
Unknown	7 (0.82)
**Index of Relative Socioeconomic Disadvantage (IRSD)**	
Quintile 1 and 2 (most disadvantaged)	78 (9.57)
Quintile 3	146 (17.15)
Quintile 4 and 5 (least disadvantaged)	659 (80.85)
Unknown	8 (0.94)
**Cancer Type (top 5)**	
Melanoma	183 (21.53)
Colon	99 (11.65)
Hodgkin lymphoma	89 (10.47)
Testicular	81 (9.53)
Thyroid	72 (8.47)

**Table 2:** Total healthcare use and associated costs over five years for adolescents and young adults with melanoma vs. other cancer types diagnosed in Queensland between 1 July 2011 and 30 June 2015.

Healthcare Type	Melanoma		Other Cancers	
	Sum Cost (AUD)	Sum Episodes	Sum Cost (AUD)	Sum Episodes
**Public**	AUD 1,452,275	326	AUD 32,304,841	4405
**Private**	AUD 660,163	256	AUD 11,958,140	4890
**ED**	AUD 227,495	371	AUD 2,032,055	2773
**MBS co-payments**	AUD 350,505	23,973	AUD 2,804,764	191,927
**PBS co-payments**	AUD 67,738	5146	AUD 643,039	53,175

**Table 3:** Median cost and health service use per patient (IQR) for melanoma and other cancers among patients.

Healthcare Type	Melanoma	Other Cancers	Melanoma	Other Cancers
	Cost per Person (Median, IQR)	Cost per Person (Median, IQR)	Episodes/Claims per Person (Median, IQR)	Episodes/Claims per Person (Median, IQR)
**Public**	AUD 2245 (0–7461)	AUD 12,167 (0–40,715) *	1 (0–2)	2 (0–6)
**Private**	AUD 0 (0–2522)	AUD 699 (0–11,190)	0 (0–1)	1 (0–3)
**ED**	AUD 393 (AUD 0–1598)	AUD 1253 (0–3339) *	1 (0–2)	2 (0–5)
**MBS co-payments**	AUD 932 (253–2526)	AUD 714 (36–4559) *	92 (58–149)	192 (108–319) *
**PBS co-payments**	AUD 185 (88–434)	AUD 516 (187–1152) *	15 (7–30)	49 (17–95) *

* Indicates a statistically significant difference (*p* < 0.05) between melanoma and other cancers.

**Table 4:** Median (IQR) number of hospital admissions and ED visits and cost per patient by sociodemographic and clinical characteristics.

Variable	Public		Private		ED	
	Median Admissions (IQR)	Median Cost (IQR) (AUD)	Median Admissions (IQR)	Median Cost (IQR) (AUD)	Median Admissions (IQR)	Median Cost (IQR) (AUD)
**Sex**						
Male	2 (0–6)	AUD 10,396 (AUD 0–49,218)	0 (0–3)	AUD 0 (AUD 0–12,058)	2 (0–5)	AUD 1256 (AUD 0–3276)
Female	2 (0–5)	AUD 14,153 (AUD 0–36,511)	1 (0–3)	AUD 1150 (AUD 0–10,598)	2 (0–5)	AUD 1253 (AUD 0–3466)
**First Nations status**						
First Nations Australians	5.5 (3–14) *	AUD 38,980 (AUD 17,882–74,125) *	0 (0–0) *	AUD 0 (AUD 0–0) *	5 (2–13.5) *	AUD 3392 (AUD 1504–8548) *
Non-First Nations Australians	2 (0–5)	AUD 11,795 (AUD 0–38,984)	1 (0–4)	AUD 1092 (AUD 0–12,558)	2 (0–4)	AUD 1145 (AUD 0–3212)
**Remoteness**						
Metropolitan	2 (0–5) *	AUD 8675 (AUD 0–30,493) *	1 (0–4)	AUD 1778 (AUD 0–12,584)	2 (0–4) *	AUD 971 (0–2792) *
Regional (inner and outer)	2 (0–4)	AUD 17,077 (AUD 2514–55,689)	0 (0–3)	AUD 0 (AUD 0–9537)	2 (1–6)	AUD 1588 (AUD 453–3879)
Remote and very remote	4 (3–9)	AUD 23,918 (AUD 20,513–132,686)	0 (0–3)	AUD 0 (AUD 0–12,519)	2 (1–6)	AUD 1319 (AUD 210–2711)
**Index of Relative Socioeconomic Disadvantage (IRSD)**						
1 and 2 (most disadvantaged)	3 (1–7) *	AUD 22,184 (AUD 10,081–57,539) *	0 (0–1)	AUD 0 (AUD 0–1989) *	3 (1–9) *	AUD 2024 (AUD 771–5923) *
3	3 (1–8)	AUD 21,224 (AUD 6255–48,937)	0 (0–1)	AUD 0 (AUD 0–2190)	2 (1–5)	AUD 1691 (391–3481)
4 and 5 (least)	1 (0–4)	AUD 8870 (AUD 0–34,977)	1 (0–4)	AUD 2340 (AUD 0–13,110)	2 (0–4)	AUD 1032 (AUD 0–3147)
**Cancer type ^^**						
Colon	2 (1–4.5) *	AUD 12,112 (AUD 6692–35,821) *	0 (0–2) *	AUD 0 (AUD 0–4768) *	3 (1–6.5) *	AUD 1655 (AUD 971–4257) *
Hodgkin lymphoma	(2–6)	AUD 13,291 (AUD 0–27,493)	1 (0–22)	AUD 4786 (AUD 0–22,247)	2 (0–4)	AUD 962 (AUD 0–2692)
Testicular	2 (0–6)	AUD 5364 (AUD 0–26,299)	0 (0–2)	AUD 0 (AUD 0–4193)	2 (0–3)	AUD 1014 (AUD 0–2423)
Thyroid	1 (0–3)	AUD 7991 (AUD 0–21,315)	1 (0–2)	AUD 4694 (AUD 0–9217)	1 (0–2.5)	AUD 406 (AUD 0–1435)
Non-Hodgkin lymphoma	3 (0–9)	AUD 21,208 (AUD 0–90,463)	2 (0–17)	AUD 2592 (AUD 0–37,608)	2 (1–6)	AUD 1926 (AUD 0–4197)

* *p* < 0.05 for overall between-group difference based on non-parametric tests (Kruskal–Wallis or Wilcoxon as appropriate). ^^ top 5 cancer diagnoses after Melanoma in AYAs in Queensland from 1 July 2011 to 30 June 2015

## Data Availability

The study utilised multiple linked administrative datasets that are not sharable and accessible to anyone who is not approved by the relevant ethics committee.

## Supplementary Materials

The following supporting information can be downloaded at https://www.mdpi.com/article/10.3390/healthcare13243302/s1. Table S1: Median cost and health service use per patient (IQR) for melanoma and other cancers among patients with non-zero service use; Table S2: Median costs and health service use per patient (IQR) by sociodemographic and clinical characteristics among those with non-zero service use.

## Abbreviations

The following abbreviations are used in this manuscript:

AYA	Adolescent and Young Adult
OOP	Out-of-Pocket
QCR	Queensland Cancer Registry
QHAPDC	Queensland Health Admitted Patient Data Collection
EDIS	Emergency Department Information System
MBS	Medical Benefits Scheme
PBS	Pharmaceutical Benefits Scheme
ASIR	Age-Standardised Incidence Rate
ASMR	Age-Standardised Mortality Rate
CNS	Central Nervous System
CCI	Charlson Comorbidity Index
HREC	Human Research Ethics Committee
AR-DRG	Australian Refined Diagnostic-Related Group
NHCDC	National Hospital Cost Data Collection
PHDB	Private Hospital Data Bureau
ED	Emergency Department
URG	Urgency Related Group
SD	Standard Deviation
AUD	Australian Dollars
