# Supplementary material for: Quantifying Cancer Healthcare Costs for Adolescents and Young Adults in Queensland, Australia
Source: Healthcare (Basel). 2025 Dec 16;13(24):3302. doi: 10.3390/healthcare13243302 (PMC12733226; doi:10.3390/healthcare13243302)
Supplement: Supplementary file 1 [file healthcare-13-03302-s001.zip › healthcare-3975552-supplementary.docx]

Supplementary Table S1: Median cost and health service use per patient (IQR) for melanoma and other cancers among patients with non-zero service use

| Healthcare Type | Melanoma | Other Cancers | Melanoma | Other Cancers |
| --- | --- | --- | --- | --- |
|  | **Cost Per Person (Median, IQR)** | **Cost Per Person (Median, IQR)** | **Episodes/claims Per Person (Median, IQR)** | **Episodes/claims Per Person (Median, IQR)** |
| Public | $6964 ($2697–15283) | $23330 (10712–57877)* | 2 (1–3) | 3 (2–8) |
| Private | $3046 (1287–6389) | $11027 (4724–31380)* | 1 (1–2) | 3 (1–13.5)* |
| ED | $1230 ($592-2644) | $2141 (988–4221)* | 2(1-4) | 3 (2–6) |
| MBS co-payments | $1224 (431–2,931) | $1,756 (291–5,636)* | 99 (62–151) | 216 (124–343)* |
| PBS co-payments | $211 (95–448) | $538 (209–1,167)* | 15 (8–31) | 52 (19–98)* |

* p < 0.05 for statistically significant group difference between melanoma and others

Supplementary Table S2: Median costs and health service use per patient (IQR) by sociodemographic and clinical characteristics among those with non-zero service use

| **Variable** | **Public** | | **Private** | | **ED** | |
| --- | --- | --- | --- | --- | --- | --- |
|  | **Median admissions (IQR)** | **Median cost (IQR) (AUD)** | **Median admissions (IQR)** | **Median cost (IQR) (AUD)** | **Median visits (IQR)** | **Median cost (IQR) (AUD)** |
| **Sex** | | | | | | |
| Male | 3(2-11) | $24966 ($8680-74858) | 4 (2-18) | $12779 ($4592-34628) | 3 (2-6) | $2096 ($1074-4203) |
| Female | 3 (2-7) | $22805 ($11963-47646) | 3 (1-10) | $9902 ($4767-27796) | 3 (2-6) | $2223 ($971-4268) |
| **First Nation status** | | | | | | |
| First Nation Australians | 5.5 (3-14)* | $38980 ($17882-74125)* | 1 (1-4) | $1907($1826-8150) | 5 (2-13)* | $3392 ($1504-8548)* |
| Non-First Nation Australians | 3 (1-7) | $22444 ($10081-56280) | 3 (1-15) | $11190 ($4767-32117) | 3 (2-6) | $2111 ($971-4119) |
| **Remoteness** | | | | | | |
| Metropolitan | 3 (1-7) | 20525 (8211-47296)* | 3 (1-15) | $10972 ($4681-28247) | 3(1-5) | $2070 (971-4069) |
| Regional (inner and outer) | 4 (2-9) | 32737 (12916-81061) | 4 (1-16) | $12588 ($4799-32995) | 3(2-7) | $2298 ($1219-4618) |
| Remote and very remote | 3 (1-5) | 16220 (20513-132686) | 3 (1-6) | $12519 ($3436-34628) | 2(1-6) | $1776 ($1000-5075) |
| **Index of Relative Socioeconomic Disadvantage (IRSD)** | | | | | | |
| 1&2 (most disadvantaged) | 3 (2-11) | $30753 ($14153-82839) | 3 (1-27)* | $15662 ($1989-47590)* | 4 (2-11) | $2556($1285-7920) |
| 3 | 4 (2-10) | $23646 ($11501-50549) | 2 (1-9) | $12519 ($2026-40019) | 4 (2-6) | $2275(1219-3481) |
| 4&5 (least) | 3 (1-7) | $22198 ($9484-56320) | 3 (2-14) | $10598 ($4916-28108) | 3 (1-6) | $2007($967-4088) |
| **Cancer type^^** | | | | | | |
| Colon | 2 (1-5)* | $13166 ($9620-40086)* | 2 (1-4)* | $7989 ($4420-17050)* | 3 (1-7) | $2080 ($1061-4604)* |
| Hodgkin Lymphoma | 5 (2-9) | $22907 ($14415-57877) | 18 (2-32) | $23060 ($6679-44102) | 5(3-8) | $4264 (2582-8581) |
| Testicular | 3 (1-9) | $12916 ($4375-48521) | 3 (1-9) | $4916 ($1975-9286) | 3(2-6) | $1903($1035-3701) |
| Thyroid | 3 (2-4) | $19820 ($10716-29723) | 2 (1-3) | $8704 ($5091-13190) | 2(1-5) | 1253 (592-3128) |
| Non-Hodgkin Lymphoma | 7 (3-12) | $57078 ($15758-113013) | 11 (6-21) | $27796 ($9731-47618) | 3 (2-7) | $2379 ($1267-6353) |

** p < 0.05 for statistically significant overall between-group difference based on non-parametric tests (Kruskal–Wallis or Wilcoxon as appropriate).
